# A special issue on contraceptive development: past, present, and future

**DOI:** 10.1093/biolre/ioaa120

**Published:** 2020-07-24

**Authors:** Martin M Matzuk, Gunda I Georg, Wei Yan

**Affiliations:** 1 Department of Pathology & Immunology and Center for Drug Discovery, Baylor College of Medicine, Houston, TX, USA; 2 Department of Medicinal Chemistry, Institute for Therapeutics Discovery and Development, School of Pharmacy, University of Minnesota, Minneapolis, MN, USA; 3 The Lundquist Institute for Biomedical Innovation at Harbor-UCLA Medical Center, Torrance, CA, USA

This special issue of *Biology of Reproduction,* focused on contraception, was galvanized by the annual *Eunice Kennedy Shriver* National Institute of Child Health and Human Development (NICHD) Contraceptive Development Meeting that was hosted by Baylor College of Medicine in Houston, Texas. The timing of the meeting (November 3–6, 2019) and the freedom that we had to interact and socialize was fortuitous because it came before the United States and the world were engulfed by the coronavirus disease 2019 pandemic, which has since limited travel and face-to-face interactions. Many of the reviews, forums, and research articles in this issue arrived in February–March 2020 at *Biology of Reproduction* before international research laboratory, foundation, organization, and government shutdowns occurred, permitting timely production of this special issue. Although there have been some delays with resubmissions because of the pandemic, we managed to hit our target for delivery of this special issue with a range of articles.

Contraceptives are designed for two main purposes: control of population growth and family planning. As of the writing of this editorial, there are nearly 7.8 billion people on our planet (see www.worldometers.info/world-population), with over one-third of the world’s population living in China (1.44 billion) or India (1.38 billion). This population expansion has had devastating effects on our environment—causing an increase in pollution, causing global warming, and eradicating species at a rate never before seen. At the same time, the unintended pregnancy rate is ~ 45% of all pregnancies worldwide, and more than half of unintended pregnancies end up in abortions (Wikipedia on “unintended pregnancy”), which is costly because of the associated morbidity and mortality. Overpopulation and high unintended pregnancy rates underscore a critical need for next-generation contraceptives, which should be effective, safe, affordable, and available to both men and women. Although research on contraceptive development has never stopped over the past several decades, enthusiasm had been dwindling largely due to limited progress and, maybe more importantly, insufficient funding and lack of interest in this topic from the pharmaceutical industry. In recent years, interest has recommenced with more academic investigators engaging in contraceptive research and the availability of more funding sources (e.g., NICHD, Bill and Melinda Gates Foundation, Male Contraceptive Initiatives, Michelson Found Animals, etc.). We, therefore, believe that it is an opportune time to publish a special issue of *Biology of Reproduction*, with a focus on the past, present, and future of contraceptive development.

The flagship review in this contraceptive special issue is written by Erwin Goldberg, who has a long history of working in this field and Daniel Johnston, Chief of the Contraception Research Branch at NICHD. The review describes the strategy for preclinical development of non-hormonal contraceptives for men and women. In particular, the review outlines key advances in “omics” and the need for target validation and presents emerging targets that are expressed exclusively in the male reproductive tract.

Because of the limited interest in male contraception by the pharmaceutical industry, it is important that novel partnerships between the biotech industry, foundations, organizations, government, and academia be developed (as presented in the Forum paper by Callahan et al. from FH360). This “Team Contraception Science” approach is described in more detail in the research article by Vahdat, Shane, and Nickels from the Male Contraceptive Institute. A successful use of this approach is the partnership between Weill Cornell Medicine scientists and the Tri-Institutional Therapeutics Discovery Institute in which they have developed potent inhibitors of soluble adenylate cyclase (ADCY10), described in the forum paper by Balbach et al.

As described in detail in the Goldberg and Johnston review, one of the first steps in the contraceptive drug discovery process is to validate that a target is required for fertility. With the advances in CRISPR/Cas9, a discovery-based approach has been chosen by the laboratories of Masahito Ikawa (Osaka University) and Martin Matzuk (Baylor College of Medicine) to quickly and simultaneously evaluate multiple targets. Following up on their recent collaborations, they have identified an additional 25 genes that are dispensable for fertility (Sun et al., Park et al., Devlin et al., and Kobayashi et al.). In parallel, they have shown that knockout of *Fam170a* (but not *Fam170b*) causes subfertility (Devlin et al.) and discovered that the absence of *Cib4* results in a spermiogenic defect (Xu et al.), the knockout of *Ssmem1* leads to an acrosomal biogenesis defect in globozoospermia (Nozawa et al.), precise deletion of *Prss55* (but not *Prss51*) causes a defect in sperm migration through the uterotubal junction (Kobayashi et al.), and the absence of the transmembrane protease encoding gene *Tmprss12* results in defects in both sperm motility and sperm migration through the uterotubal junction (Lasarati et al.). Likewise, other groups have been working on similar fertility-related genes. Salicioni et al. (University of Massachusetts-Amherst) have reviewed the testis-specific serine kinase family.

Multipurpose technologies (MPTs) are being developed as anti-infectives and contraceptives to block sperm from reaching the oocyte in the female reproductive tract. This topic is reviewed by Anderson et al. (Boston University School of Medicine) and discussed in a Forum article by Achilles et al. (CAMI Health). There is an additional research article presented by Hemmerling, Christopher, and Holt (CAMI Health) that describes a roadmap for development of MPTs for non-hormonal contraception and prevention of infection by HIV and other STDs. Weitzel, North, and Waller (Yaso Therapeutics) present an update on polyphenylene carboxymethylene as a non-hormonal drug in a vaginal gel as a first step toward a topical, easy to use produce for both contraception and protection from sexually transmitted diseases. Relevant to this topic, Calla et al. (Stanford University) included a research paper and a forum article on steroids, vaginal barrier protection, and sexually transmitted diseases.

Small molecule contraceptives are being developed through a targeted approach. These include small molecules that target the P2X1-purinoceptor (Mathiew et al., Monash University) and the MEIOB-SPATA22 complex (Xu et al., University of Pennsylvania). In addition, there are multiple targets that have involved collaborations with Gunda Georg’s group at the University of Minnesota. These include targeting of the ATP1A4 Na^+^/K^+^ ATPase (Syeda et al., with collaborators at the University of Kansas Medical Center) and cyclin dependent kinase 2 (Faber et al.). These collaborations have also extended to an oocyte-specific kinase target, WEE2, with Hanna et al. at the Oregon National Primate Research Center and Oregon Health and Science University. Studies from the Wolgemuth laboratory at Columbia University describe the phenotypic effects of disruption of retinoid signaling in the testis (research paper by Chung et al.) and the state of retinoic acid receptor alpha antagonists for male contraception (review article by Noman et al.).

A novel approach to contraception in the female reproductive tract is presented by Winuthayanon and colleagues at Washington State University. Serine proteases are important in semen liquefaction, and the blockade of this process is a unique non-hormonal contraceptive method, described in a research paper by Barton et al. and in a review by Anamthathmakula and Winuthayanon.

Lastly, an important research paper by Li et al. (Baylor College of Medicine) describes the metabolism of the male-specific reversible contraceptive molecule, JQ1, which targets the testis-specific bromodomain protein, BRDT. Mauck (Daré Biosciences Inc.) and Vincent (University of Texas Medical Branch) reviewed the use of the postcoital test for evaluation of vaginal-based contraceptives.

Given the recently resumed enthusiasm for the development of next-gen contraceptives for women and the first ever male pills for men, we hope that the papers in this special issue will serve as a catalyst to excite more investigators and investors to expedite the drug discovery and development process. We also welcome feedback on this special collection of papers on contraceptive development.

